# What Impact does An Angry Context have Upon Us? The Effect of Anger on Functional Connectivity of the Right Insula and Superior Temporal Gyri

**DOI:** 10.3389/fnbeh.2016.00109

**Published:** 2016-06-06

**Authors:** Viridiana Mazzola, Giampiero Arciero, Leonardo Fazio, Tiziana Lanciano, Barbara Gelao, Teresa Popolizio, Patrik Vuilleumier, Guido Bondolfi, Alessandro Bertolino

**Affiliations:** ^1^Department of Mental Health, Psychiatry de Liaison, University Hospitals of GenevaGeneva, Switzerland; ^2^Laboratory for Behavioral Neurology and Imaging of Cognition, Department of Neurology, University Hospital and Department of Neuroscience, Medical School University of GenevaGeneva, Switzerland; ^3^Institute of Post-Rationalist Psychology IPRARome, Italy; ^4^Department of Basic Medical Sciences, Neuroscience and Sense Organs, University of Bari “Aldo Moro”Bari, Italy; ^5^Department of Education, Psychology, Communication, University of BariBari, Italy; ^6^Department of Neuroradiology, “Casa Sollievo della Sofferenza” IRCCSSSan Giovanni Rotondo, FG, Italy; ^7^Department of Psychiatry, University of GenevaGeneva, Switzerland

**Keywords:** anger, right insula, superior temporal gyri, social affective engagement, dynamic causal modeling

## Abstract

Being in a social world requires an understanding of other people that is co-determined in its meaning by the situation at hand. Therefore, we investigated the underlying neural activation occurring when we encounter someone acting in angry or joyful situation. We hypothesized a dynamic interplay between the right insula, both involved in mapping visceral states associated with emotional experiences and autonomic control, and the bilateral superior temporal gyri (STG), part of the “social brain”, when facing angry vs. joyful situations. Twenty participants underwent a functional magnetic resonance imaging (fMRI) scanning session while watching video clips of actors grasping objects in joyful and angry situations. The analyses of functional connectivity, psychophysiological interaction (PPI) and dynamic causal modeling (DCM), all revealed changes in functional connectivity associated with the angry situation. Indeed, the DCM model showed that the modulatory effect of anger increased the ipsilateral forward connection from the right insula to the right STG, while it suppressed the contralateral one. Our findings reveal a critical role played by the right insula when we are engaged in angry situations. In addition, they suggest that facing angry people modulates the effective connectivity between these two nodes associated, respectively, with autonomic responses and bodily movements and human-agent motion recognition. Taken together, these results add knowledge to the current understanding of hierarchical brain network for social cognition.

## Introduction

In our everyday lives we constantly make sense of other people and ourselves in order to coordinate with our social world. How exactly we manage to do this in various different circumstances is less clear. Indeed, there are many levels of complexity implied in understanding others, depending, for instance, on the amount of information at our disposal, the role we play (e.g., as a passive viewer or as an interactant), the historical background of the ongoing social experience, as well as the impact of implications and consequences of our behavior.

Research concerned with social perception has been focused on the information-processing “machinery” by which individuals attempt to understand others’ behaviors (McArthur and Baron, 1983; Baldwin, 1992). More specifically, such research reveals what it is in a person’s movements, gestures, voice, and facial appearance that communicates to us that person’s momentary intentions and emotional states. This ability to recognize, manipulate, and behave with respect to socially relevant information relies on neural systems that process of social signals. Among other brain areas, the bilateral superior temporal gyri (STG) are largely involved when processing a diverse array of social cues like others’ faces, voices and gestures as well as contextual information (Allison et al., 2000; Adolphs, 2003; Singer et al., 2004; Paulus et al., 2005; Xu et al., 2009). Neuroimaging evidence shows also that these areas are highly implicated in psychiatric disorders with significant impairments of social cognition and social functioning as in autism spectrum disorder and schizophrenia (Cheng et al., 2015; Green et al., 2015). We clearly make great use of such “social” information in our daily life to have an impression about people and situations, for example, when deciding whom to ask for directions or whom to steer clear of in the subway. However, current research does not provide us with an accurate picture of whether and how the emotional context in which we are interacting has an impact upon us.

Indeed, the contribution made by the emotional contexts in which we see others behave has not been thoroughly investigated. Yet, we encounter others doing this or that in worldly situations, and our way of being together and understanding each other is co-determined in its meaning by the situation at hand (Gurwitsch, 1979). Furthermore, action has a specific revelatory quality. Indeed, acting implies the disclosure of who is acting (Arendt, 1958). Talking on the phone, as well as grasping a glass in a certain way, will disclose her/him in this or that way, e.g., annoyed, inappropriate, amused or absorbed. Emotional context influences are also important insofar as they refer to the ways by which single items are affected by surrounding scene elements and the way in which global scene characteristics affect sense making processing. Indeed, the emotional context in which an action is seen affects the neural underpinnings of movement control in a different way (Mazzola et al., 2013).

Anger is largely seen as an interpersonal emotion (Siegel, 1986; Smith and Lazarus, 1993; Fehr et al., 1999). For instance, it cannot be fully understood when removed from the social context in which it occurs and situational characteristics also affect the occurrence of anger related behaviors (Averill, 1983; Kuppens et al., 2004). In addition, its expression in a social context can be used strategically in interaction (Gneezy and Imas, 2014). On the other hand, anger has detrimental consequences on people. Being exposed to anger can have a significant effect on biological regulatory systems, also early in life (Pollak et al., 2005). It is also well known that the sympathetic response of the autonomic nervous system elicited by anger has a significant impact on cardiac arrhythmogenesis (Lampert et al., 2002; Craig, 2005; Suls and Bunde, 2005; Taggart et al., 2011; Steptoe and Kivimäki, 2012; Suls, 2013; Mostofsky et al., 2014; Buckley et al., 2015; Lampert, 2016). Several studies have established a relationship between cardiovascular sympathethic control and the right insular cortex (Oppenheimer et al., 1992; Critchley et al., 2000; Oppenheimer, 2006). Indeed, the viscerotopic sensory representation in the insula and its input and output connections enable the insula to play a primary role in brain-heart interactions (Nagai et al., 2010).

Assuming that in social situations the emotional context can make a difference (Mazzola et al., 2013), the underlying neural activation occurring when we encounter someone acting in an “angry” context remains unknown. Therefore, here we investigated the dynamic interplay between the right insula both involved in visceral aspect associated with emotional experiences and in the autonomic control of cardiovascular arousal (Critchley et al., 2000, 2005; Critchley, 2009), and the bilateral STG broadly involved in social perception and contextual information (Allison et al., 2000; Adolphs, 2003; Singer et al., 2004; Paulus et al., 2005; Xu et al., 2009), when facing an angry situation.

To address this question, we employed our previous functional magnetic resonance imaging (fMRI) task on a new independent sample of twenty participants (Mazzola et al., 2013). Participants watched two runs of video clips of an actor grasping an object performing the same gesture but with different emotional expressions (neutral, joyful, or angry). In order to set two distinct emotional contexts, one run included only neutral and joyful expressions, while the other featured only neutral and angry expressions. Our design allowed us to investigate the brain activity elicited by the observation of someone acting in angry and joyful situations, while keeping action kinematics constant.

To better understand the right insula and STG engagement during the angry situation, we employed two different but complementary methods for investigating brain connectivity. First, we used a psycho-physiological interaction (PPI) analysis to examine changes in the functional connectivity of the right insular region under the angry session (angry vs. joyful session). Next, we examined whether anger modulated the connections within our regions of interest using dynamic causal modeling (DCM) analyses. For this purpose, we created 14 possible DCMs, and we used Bayesian model comparison to obtain statistical estimates of which model offered the optimum balance between simplicity and fit to the data (Penny et al., 2004).

## Materials and Methods

### Ethics Statement

The present study was approved by the local committee. Informed written consent was obtained from all participants before participation.

### Participants

Twenty participants were enrolled (8 females; mean age 27; standard deviation [SD] 3.03, range = 23–33). Exclusion criteria included a history of drug or alcohol abuse, previous head trauma with loss of consciousness, pregnancy, and any significant medical or psychiatric conditions as evaluated with the Structured Clinical Interview (SCID). The present study was approved by the local institutional review board.

### fMRI Experimental Paradigm

The fMRI task employed was described in detail in our previous study (Mazzola et al., 2013). The fMRI session consisted of two successive scanning runs with an event-related design. Each run included one emotion (joy or anger) and consisted of four experimental view conditions. All visual stimuli consisted of video clips of 1.3 s. There were four different video conditions in each run, showing the following actions: the trunk with an arm grasping an object on a table (grasping alone), a person with a neutral facial expression grasping an object on a table (neutral grasping), a person with a joyful or angry facial expression grasping an object on a table (joyful grasping in the joyful run or angry grasping (AG) in the angry run), and a joyful or angry dynamic facial expression without any grasping action (joyful face in the joyful run or angry face or in the angry run). The recording and editing of videos were made using the Blue Screen technique in order to superimpose on the same trunk different emotional facial expressions. This procedure allowed us to obtain stimuli with the same kinematics of grasping behavior across different emotional conditions. Two professional actors, a female and a male, were enrolled as models for the videos. The everyday objects to be grasped were put on a table, e.g., phone, pen, keys, bottle, cup, and glass.

To circumvent any motor interference, we used a passive viewing task and participants were instructed to remain still without performing any movement, and to avoid any imitation or mental imagery of the actions shown, and to carefully look at the video clips in order to get involved. The presentation order of the two runs was counterbalanced across subjects.

### fMRI Data Acquisition and Analyses

Three-dimensional images were acquired using a T1-weighted SPGR sequence (TR/TE/NEX = 25/3/1; flip angle 6°; matrix size 256 × 256; FOV 25 × 25 cm) with 124 sagittal slices (1.3 mm thick, in-plane resolution of 0.94 × 0.94). fMRI data were acquired on a 3T GE (General Electric, Milwaukee, WI, USA) MRI scanner with a gradient-echo echo planar imaging (EPI) sequence and covered 26 interleaved axial slices (5 mm thick, 1 mm gap), encompassing the entire cerebrum and the cerebellum (TR 2; FOV 24 cm; matrix, 64 × 64, a voxel size of 3.75 × 3.75 × 5 mm). For each scan, a total of 285 EPI volume images were acquired.

#### Preprocessing

Data were preprocessed and analyzed using statistical parametric mapping SPM8 (Wellcome Department of Cognitive Neurology, London, UK), implemented in MatLab 7.8 (MathWorks^TM^). A fixed-effect model at a single-subject level was performed to create images of parameter estimates, which were then entered into a second-level random-effects analysis. For each subject, functional images were first slice-timing corrected, using the middle slice acquired in time as a reference, and then spatially corrected for head movement, using a least-squares approach and six-parameter rigid body spatial transformations. High-resolution anatomical T1 images were coregistered with the realigned functional images to enable anatomical localization of the activations. The two runs were then concatenated. Structural and functional images were spatially normalized into a standardized anatomical framework using the default EPI template provided in SPM8, based on the averaged-brain of the Montreal Neurological Institute and approximating the normalized probabilistic spatial reference frame of Talairach and Tournoux (1998). Functional images were spatially smoothed with a three-dimensional Gaussian filter (10 mm full-width at half-maximum (FWHM)). The time series was temporally filtered to eliminate contamination from slow drift of signals (high-pass filter, 128 s) and corrected for autocorrelations using the AR(1) model in SPM8.

#### Statistical Analyses

We performed two parallel but identical statistical analyses on the functional data for the whole-brain and cerebellar normalized images. Four event-types were defined per subject per scanning run, corresponding to each condition of interest. In the joyful run, the conditions of interest were: grasping alone, neutral grasping, joyful grasping, joyful face. In the angry run, the conditions were: grasping alone, neutral grasping, AG, angry face. Eight contrast images corresponding to these conditions were then created using one-sample *t*-tests in all subjects and then entered at the second level into a repeated-measures 2 × 4 ANOVA (flexible factorial design implemented in SPM8). Because of our strong *a priori* hypothesis, results in the right insula and bilateral STG were corrected for multiple comparisons with family-wise error (FWE) correction at *p* < 0.05 applied on the activated clusters in an *F*-test, with the volume of interest defined by the corresponding anatomical region in the WFU Pickatlas^1^. We also reported regions that were not predicted *a priori* but survived a threshold of *p* < 0.05 cluster-level FWE corrected. Cerebral MNI coordinates were converted to the Talairach coordinate system by icbm2tal^2^. Anatomic and Brodmann areas labeling of cerebral activated clusters was performed with the Talairach Daemon database^3^. In order to check whether our prior findings were replicated (Mazzola et al., 2013), a small-volume correction was applied based on our previous main results using a sphere with a radius of 10 mm centered around coordinates from previously published studies [MNI coordinates: supplementary motor area (*x* = −7, *y* = 27, *z* = 65), thalamus (*x* = −14, *y* = −11, *z* = 10), cerebellum HV (*x* = 18, *y* = −38, *z* = −21)].

#### Cerebellar Normalization

We used a separate normalization process for data from the cerebellum. The registration between individuals and MNI space is suboptimal in the cerebellum when using a standard whole-brain normalization process (Diedrichsen et al., 2009). Because cerebella vary relatively little between individuals compared with the cortical landmarks used for whole-brain normalization, it is possible to achieve a much better registration by normalizing the cerebella separately. Moreover, precise spatial registration is important because cerebellar structures are small compared to cortical structures. To this aim, we used the SUIT toolbox (Diedrichsen et al., 2009) for SPM8 allowing us to normalize each individual’s structural scan to an infratentorial template, and then used the resulting deformation maps to normalize the cerebellar sections of each person’s functional images. The SUIT toolbox has the additional advantage that coordinates can be adjusted from MNI space to the corresponding coordinates on the unnormalized Colin-27 brain, which is described anatomically in a cerebellar atlas. We used this feature to identify anatomical regions within the cerebellum.

#### PPI Analyses

Region-specific BOLD time-series were extracted as the first eigenvariate of all significant voxels within a 6 mm radius sphere centered on each participant’s local maxima in right insula and bilateral STG, as obtained from the respective *F*-contrast angry vs. joyful session [MNI coordinates: right insula (*x* = 38, *y* = −7, *z* = −5), right STG (*x* = 50, *y*= −37, *z* = 20), left STG (*x* = −56, *y* = −33, *z* = 20)]. First, we used PPI to examine changes in connectivity of the right insula as a seed region under the angry context (anger vs. joyful session). A general linear model was computed using three regressors: a physiological regressor (the time-series in the ROI), a psychological regressor (angry session—joyful session), and a PPI term, calculated as the cross-product of the previous two terms. These time-series were then mean centered, high-pass filtered, and deconvolved. Subject-specific contrast images were then entered into a random effects analysis using paired *t*-tests (*p* < 0.01 whole-brain FWE corrected).

#### DCM Analyses

**Figure 1:**
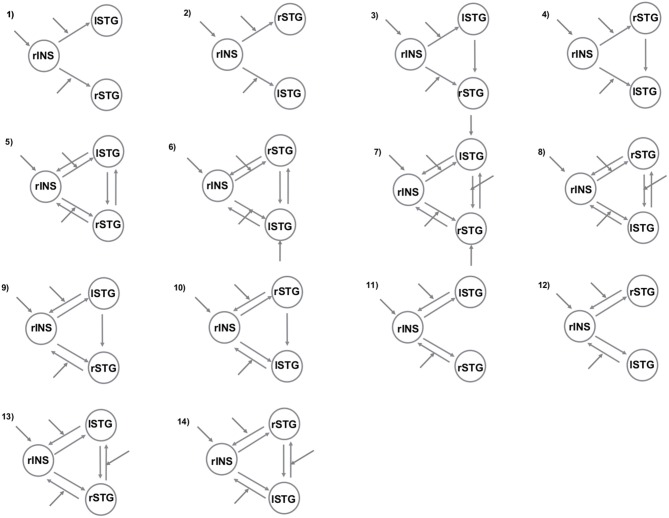
**The models tested with Bayesian model selection (BMS).** The patterns of modulatory influences of anger on connectivity among right insula (rINS), left STG (lSTG), and right STG (rSTG).

To understand further the effective connectivity between our ROIs, we used DCM (Friston et al., 2003). More specifically, the DCM explains regional effects in terms of the changing patterns of connectivity among regions according to the experimentally induced contextual modulation of connection strengths. Accordingly, we examined the mutual influences within this brain network involved in the angry session using DCM. Our DCM design matrices comprised a regressor modeling of the sensory input (all conditions in angry session), and a regressor modeling of the effect of the anger (AG condition). After estimation, the individual models were compared using random-effects Bayesian model selection (RFX BMS) to determine the model with the overall highest evidence (Stephan et al., 2009). We report each model’s exceedance probability, which reflects how a model is compared with the competing models. In all models, the right insula received all the conditions of the angry run as a driving input. In addition, all the regions were connected to one another with all combinations of either 2 or 3 bidirectional connections consistent with anatomical evidence for connections between these regions in human beings (Augustine, 1996; Cloutman et al., 2012). In order to answer our research questions, we varied the position of left and right STG region connected to the right insula and the regions and connections that were affected by the anger grasping across the models (Figure 1). Both analyses (PPI and DCM) were performed based on the same seed regions.

### Questionnaires and Correlation Analyses

In order to evaluate the subjective tendencies to anger response and the subjective sensibility to bodily responses, the participants completed the State Trait Anger Expression Inventory (STAXI; Spielberger, 1989), and the Body Perception Questionnaire (BPQ; Porges, 1993). Before the scanning session, each participant completed also the State-Trait Anxiety Inventory (STAI; Spielberger, 1983) to evaluate their current state of anxiety in addition to the other questionnaires. Questionnaire data were analyzed with SPSS22 (Inc., 2009, Chicago, IL, USA). To test for the gender effect, a two sample *t*-test between males and females were performed on the STAXI measures (two tailed, *p* < 0.05). In order to test for any significant correlation between STAXI and the BPQ scores, we performed Spearman’s rho correlation analyses (two tailed, *p* < 0.05).

## Results

### Self-Report Questionnaires

Table 1 shows sample means and SD of all individual difference scale scores. No significant gender effects were found for the STAXI measures. In the current sample of healthy participants STAXI and BPQ scores fell within the normative range (not shown).

**Table 1:** **Descriptive statistics**.

Scale	Mean	SD
State-anger	15.74	5.52
Trait-anger	21.32	5.30
Trait-anger-temperament	7.21	2.62
Trait-anger-response	10.11	2.33
Anger-in	20.32	3.15
Anger-out	15.84	3.64
Anger-control	25.95	4.62
Anger expression	26.21	8.47
Awareness of bodily processes	2.75	0.55
Stress response	2.52	0.66
Autonomic nervous system reactivity	1.52	0.30

We explored any correlations between STAXI and the BPQ. The autonomic nervous system reactivity showed a very strong positive correlation with scores on the trait anger-temperament (*r* = 0.70 *p* < 0.01), while a negative one less strong with the control of anger expression scores (*r* = −0.478 *p* < 0.04).

According to the STAXI results (see Table 1), our participants were more prone to express unprovoked anger than to control and suppress it. Additionally, the strong correlation between the trait anger-temperament and the autonomic nervous system reactivity suggests the sensitivity to the bodily perception may also contribute to subjective tendencies to anger response.

### Neuroimaging Results

#### Whole-Brain General Linear Model Analyses

The angry session revealed greater activation in the left posterior insula and the left hippocampus. The AG > joyful grasping contrast revealed the same clusters (*p* < 0.05 FWE cluster-level corrected; see Table 2). In contrast, no activation reached the statistical significance in the reverse contrasts. On the other hand, the main effect of grasping observation (grasping alone > faces alone, pooled across both emotion sessions) showed significant activations in the left inferior and right middle occipital gyrus, in the bilateral middle frontal gyrus and left inferior frontal gyrus/pars triangularis, as well as in the left thalamus and the left middle temporal gyrus (*p* < 0.05 FWE corrected; see Table 2). In the reverse contrast (faces alone > grasping alone, pooled across both emotion sessions) only the right middle temporal gyrus survived (*p* < 0.05 FWE corrected; see Table 2). According to our previous study (Mazzola et al., 2013), to specifically test for the effect of the context, we carried out an interaction analysis between grasping accompanied with a neutral face and grasping alone in the two different emotion runs (i.e., [neutral grasping—grasping alone] in angry runs vs. [neutral grasping—grasping alone] in joyful runs; and vice versa). We partially replicated the previous findings. Indeed, the left superior frontal gyrus/supplementary motor area and right HV showed a greater activation in neutral grasping in the angry context at a lower threshold (*p* < 0.005 uncorrected; Table 2). The thalamic activity was not replicated.

**Table 2:** **Whole-brain general linear model, *P* < 0.05 family-wise error (FWE)**.

	MNI coordinates				
Region	*x*	*y*	*z*	Ke	*Z* scores
**Angry > Joyful session**
Left posterior insula	−37	−7	5	15	5.10
Left hippocampus	−37	−30	−5	3	4.50
**Grasping > Faces**
Left inferior occipital gyrus	−48	−71	−5	1679	Inf
Right middle occipital gyrus	48	−82	5	1598	Inf
Left middle frontal gyrus	−26	4	50	195	7.07
Right middle frontal gyrus	38	42	40	246	6.41
Left inferior frontal	−56	15	30	26	5.50
Gyrus/pars triangularis
Left thalamus	−18	−30	0	13	5.25
Left superior medial gyrus	1	42	25	23	4.99
Left middle temporal gyrus	−63	−11	−5	8	4.93
Left thalamus	1	−7	10	12	4.71
**Faces > Grasping**
Right middle temporal gyrus	57	−41	−5	26	5.90
Right HVI lobule*	32	−58	−27	1486	3.89
Right VIIa crus I*	32	−74	−23		
**Interaction analysis between grasping and neutral grasping**
Left SMA BA6°	−14	30	50	11	3.08
Right cerebellum HV°	18	−28	−43	20	3.00

*p < 0.001 °p < 0.005.

#### ROI Analyses

**Figure 2:**
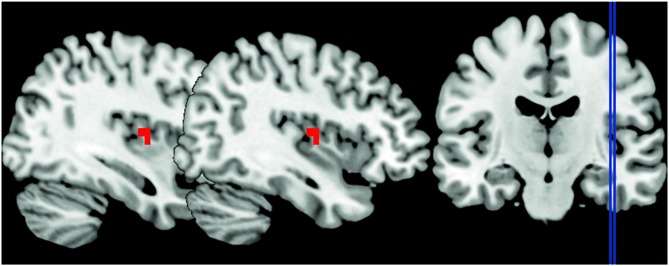
**The peak signal changes that occurred in the right posterior insula in angry session compared to the joyful one, identified at *P* < 0.05 family-wise error (FWE) cluster-level corrected *P* = 0.011, peak coordinates *x* = 38, *y* = −7, *z* = 10**.

To address whether the processing of anger impacts on the right insula activity, we tested the main effect of the emotional contexts. We found greater activation in the right insula when participants watched the angry session compared to the joyful one (*p* < 0.05 FWE cluster-level corrected *P* = 0.011; see Table 3; Figure 2). In contrast, no activation reached statistical significance in the joyful session when compared to the angry one. We also evaluated which brain regions were more sensitive to the angry vs. joyful people while grasping objects (AG > joyful grasping; *p* < 0.05 FWE cluster-level corrected *P* = 0.001; see Table 3). Activity in the same area was found. Consistent with our *a priori* hypothesis, these results added support to the view that anger of others enhances activity within the right insula.

**Table 3:** **ROIs and PPI analyses, *P* < 0.05 FWE cluster-level corrected**.

	MNI coordinates				
Region	*x*	*y*	*z*	Ke	*Z* scores
**Angry > Joyful run**
Right posterior insula	38	−7	10	6	4.50
**Angry grasping > Joyful grasping**
Right posterior insula	34	−11	10	15	4.80
**PPI: seed in the right Insula**
***P* < 0.01 FWE corrected**
Left superior temporal gyrus	−56	−7	15	47	5.60
Right superior temporal gyrus	57	−18	0	37	5.44
Left superior frontal gyrus	−33	57	20	27	5.43
Right postcentral gyrus	31	−33	70	40	5.38
Left precuneus	1	−37	50	69	5.30
Right inferior frontal gyrus	34	−14	15	40	5.26

Next, we explored the changes in connectivity associated with these angry context effects focused on our set of *a priori* ROIs: right insula and bilateral STG. First, we used PPI to examine changes in the functional connectivity of the right posterior insula as a seed region under the angry context. PPIs results showed that this region was functionally coupled with the bilateral STG, the left superior frontal gyrus, the right postcentral gyrus, the left precuneus, and the right inferior frontal gyrus/BA47 during angry vs. joyful grasping (*p* < 0.01 FWE corrected; Table 3). No significant effects were found for the joyful vs. AG PPI.

**Figure 3:**
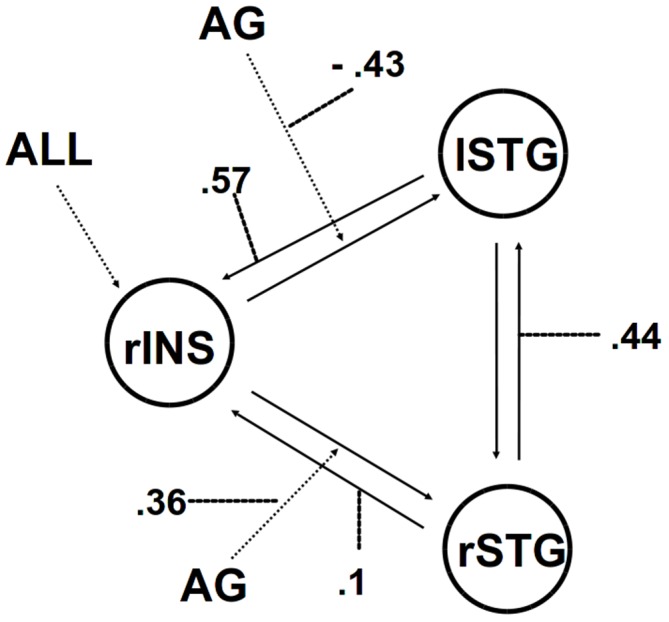
**The winning model.** Solid lines indicate fixed connectivity. Dashed arrows indicate input into the system by condition (ALL) and modulations on the given connection by angry grasping (AG). Dotted lines indicate the values of modulatory parameters.

We then applied DCM analysis to investigate the causal architecture of neural effective connectivity that may underlie the processing of others’ anger within our regions of interest. The optimal model was the one showing a negative modulation by AG on the forward connection from the right insula to the left STG (Figure 3). The exceedance probability of this model was 0.837 (Table 4) and there was an expectation of posterior probability of one. The next best model, model 1, had an exceedance probability of 0.029. This result indicated that anger did not modulate the forward connection between the right insula and the bilateral STG. The modulatory effect of AG on the forward connection reveals noteworthy findings. First, while looking at the AG, the forward connection from the right insula to the left STG is suppressed. The sign change indicates that the connection is attenuated by anger. On the other hand, the modulatory effect of AG on the forward connection from the right insula to the right STG was positive. The effect of looking at angry people increased the ipsilateral connectivity between the right insula and the right STG, while decreased the contralateral connectivity with the left STG.

**Table 4:** **DCM models exceedance probability**.

	**M1**	**M2**	**M3**	**M4**	**M5**	**M6**	**M7**
Ex. prob.	0.0288	0.0283	0.0133	0.0145	0.8374	0.0069	0.0071

	**M8**	**M9**	**M10**	**M11**	**M12**	**M13**	**M14**

Ex. prob.	0.0073	0.0073	0.0071	0.0137	0.0134	0.0075	0.0075

## Discussion

In this study we investigated insula and STG connectivity while participants watched video clips of actors grasping objects in joyful and angry contexts. Our research question concerned whether the angry context affected the insula reactivity and its functional connections to the bilateral STG.

Consistent with our prediction, the right posterior insula was significantly engaged by anger. Our results showed greater right posterior insular activity in the angry context as well as in the AG condition. Moreover, we also found a significant engagement in the left posterior insula and the left hippocampus. These results are in line with previous studies which reported the engagement of the insula when perceiving angry or aversive stimuli (Straube et al., 2004; Simmons et al., 2006; Paulus et al., 2010).

Accordingly, we performed PPI analyses with a seed region placed in the right posterior insula in order to explore possible changes in functional connectivity associated with the angry context effect. The right insula exhibited a strong positive connectivity with bilateral STG selectively only in the AG condition. Compared with PPI analysis, DCM analysis estimates effective (causal) connectivity between multiple brain regions, providing a more systematic and nuanced directional depiction of the neural network in question. Indeed, the winning model revealed that the modulatory effect of AG increased the ipsilateral forward connection between the right insula and the right STG while suppressing the contralateral one. This result suggests that the reaction to others’ anger shifts the bi-directional connection between insula and STG from a bilateral to an ipsilateral one.

Taken together these findings indicate that the response to an angry situation modifies a functional coupling between the right insula and the bilateral STG. The DCM analysis showed that the AG significantly affects the information flow within the three brain regions. This reveals a direct influence of the right insula on the right STG, and suggests an underlying neural mechanism for prioritized processing of anger-related situation. On one hand, it is well known that the posterior insula is essential for awareness of visceral states and bodily movements and therefore for coordinated action (Farrer et al., 2003; Cauda et al., 2012; Christopher et al., 2014). The present findings seem also to suggest that the emotional content of a situation may modulate this process. On the other hand, evidence shows that, being bi-directionally connected to the insula (Augustine, 1996; Cloutman et al., 2012), the STG activate it bilaterally during the assessment and action selection stage for establishing a response (Paulus et al., 2005). Indeed, STG involvement can be triggered automatically by socially salient stimuli (Singer et al., 2004) as well as being involved in visuomotor integration and visual analysis of the other’s actions and in spatial awareness (Karnath and Baier, 2010). We might speculate an interaction effect between right ipsilateral insula-STG connectivity and the right-lateralized ventral attention network (Corbetta and Shulman, 2011). Indeed, the right STG plays a role in this attention network, since it is involved in involuntary (stimulus-driven) orienting which directs attention to salient events (Ellison et al., 2004). Thus, our findings indicate that facing angry people affects the social contextual information processing leading to a greater visceral involvement.

Some studies showed the involvement of the amygdala during the processing of angry faces (Carré et al., 2012; Passamonti et al., 2012), though these studies point toward additional variables related to amygdala reactivity to angry facial expressions, such as trait anxiety. Our results consistently suggest that the insula also plays a primary role in response to anger. In addition, scores on STAXI indicate that our participants were more prone to express unprovoked anger than to control and suppress it. Moreover, the interoceptive sensibility strongly correlated with the disposition to express anger without provocation. According to Williams et al. (2001), the tendency towards quick, unprovoked (or minimally provoked) anger may be toxic to the cardiovascular reactivity. Unfortunately, besides the strongly positive correlation between the angry temperament and the sensibility to the autonomic bodily reactions, we were not able to establish to what extent subjective dispositions contributed to the insular activity reported in the present study. Indeed, it is well known that interindividual variability in affective and cognitive dispositions modulate the insula response (Cheng et al., 2007; Gu and Han, 2007; Mazzola et al., 2010). They may also play an important role in accounting for the partial replication we found in our previous findings (Mazzola et al., 2013). Yet, differences in dispositional affect influence regional brain activity associated with emotion processing (Hamann and Canli, 2004). Further investigation is required to better control for individual differences.

Another limitation of the present study concerns the lack of physiological measures concerning subjective autonomic activity. Because of this, we were not able to take into account any difference in the bilateral insula reactivity in terms of the left-sided parasympathetic and right-sided sympathetic activity (Craig, 2005). Although we did not demonstrate a different impact of trait anger on cardiovascular responses in angry vs. joyful contexts, the results we found provide some indirect evidence about the potential brain mechanisms linking anger to neurogenic heart diseases. Indeed, they suggest that a subjective response to anger can enhance the insula reactivity and affect its causal connectivity.

In conclusion, our results highlight that encountering people grasping objects in an angry situation elicited a socially affective engagement that affects the right insular reactivity. Moreover, the fact that AG shifted the bi-directional connection between insula and STG from a bilateral to an ipsilateral one indicates an emotional modulation of effective connectivity in two nodes associated, respectively, with autonomic responses and bodily movements (Farrer et al., 2003; Karnath and Baier, 2010) and human-agent motion recognition (Han et al., 2013). We argue that being affectively engaged influences the social information processing revealing an underlying neural mechanism for prioritized processing of anger-related situation. We speculate that it would impact on behavior leading toward a more automatic focus on a set of possible actions oriented to react, while leaving in the background other sets of possibilities (Frijda, 1986; Arciero and Bondolfi, 2009; Mazzola et al., 2013). Furthermore, showing the primary role played by the insula in response to anger, the present results may provide a new insight into addressing research on the neural underpinnings of the putative mechanisms underlying emotional triggers and neurogenic heart diseases (Samuels, 2007; Edmondson et al., 2013). Our findings also lead us to argue that research into social brain networks can be usefully linked to action understanding, as well as in terms of social affective engagements triggered by human encounters in a social world (Gurwitsch, 1979).

## Author Contributions

VM contributed to the design of the experiment, analysis of the data and the editing of the manuscript, GA, BG contributed to the design of the experiment and the editing of the manuscript, LF, TL, BG, TP contributed to the acquisition of the data, PV and AB contributed to the editing of the manuscript. All authors listed, have made substantial, direct and intellectual contribution to the work, and approved it for publication.

## Conflict of Interest Statement

The authors declare that the research was conducted in the absence of any commercial or financial relationships that could be construed as a potential conflict of interest.

## Footnotes

^1^http://fmri.wfubmc.edu/cms/software#PickAtlas

^2^http://brainmap.org/icbm2tal/

^3^http://www.talairach.org/
